# Transport and Barrier Functions in Rainbow Trout Trunk Skin Are Regulated by Environmental Salinity

**DOI:** 10.3389/fphys.2022.882973

**Published:** 2022-05-13

**Authors:** D. Doyle, B. Carney Almroth, K. Sundell, N. Simopoulou, H. Sundh

**Affiliations:** Department of Biological and Environmental Sciences, University of Gothenburg, Gothenburg, Sweden

**Keywords:** ussing chamber, V-ATPase, tight junction (TJ), fish skin, wound healing

## Abstract

The mechanisms underpinning ionic transport and barrier function have been relatively well characterised in amphibians and fish. In teleost fish, these processes have mostly been characterised in the gill and intestine. In contrast, these processes remain much less clear for the trunk skin of fish. In this study, we measured barrier function and active transport in the trunk skin of the rainbow trout, using the Ussing chamber technique. The effects of epithelial damage, skin region, salinity, and pharmacological inhibition were tested. Skin barrier function decreased significantly after the infliction of a superficial wound through the removal of scales. Wound healing was already underway after 3 h and, after 24 h, there was no significant difference in barrier function towards ions between the wounded and control skin. In relation to salinity, skin permeability decreased drastically following exposure to freshwater, and increased following exposure to seawater. Changes in epithelial permeability were accompanied by salinity-dependent changes in transepithelial potential and short-circuit current. The results of this study support the idea that barrier function in rainbow trout trunk skin is regulated by tight junctions that rapidly respond to changes in salinity. The changes in transepithelial permeability and short circuit current also suggest the presence of an active transport component. Immunostaining and selective inhibition suggest that one active transport component is an apical V-ATPase. However, further research is required to determine the exact role of this transporter in the context of the trunk skin.

## Introduction

The mucosal barriers of fish include the skin, gill and intestine. As these membranes are the first point of contact between the animal and the environment, their integrity is crucial for fish health and welfare (Segner et al., 2012; Rombout et al., 2014). The mucosal barriers are essential to separate the internal environment of the fish from the external environment. In this respect, they allow the fish to osmoregulate by limiting passive ion and fluid diffusion. Further, some mucosal epithelia such as those of the gill, kidney, and intestine are sites of active ionic transport (Loretz, 1995; Marshall, 2002; Takvam et al., 2021). This combination of barrier function and active transport is vital to maintain ion homeostasis, as fish are constantly exposed to osmotic pressures that challenge the internal environment. In addition to facilitating ion homeostasis, the mucosal surfaces provide an important barrier to prevent pathogens from entering the fish and causing infection (Gomez et al., 2013). The contribution of the intestine and gills to ion transport and barrier function have been relatively well described (Evans et al., 2005; Grosell, 2010; Whittamore, 2012). In comparison, less is known about these processes in the skin.

Fish skin consists of two main layers, an outer epidermis and an inner dermis with scales attached. While both the epidermis and dermis function in concert as a protective barrier for internal organs, muscles, nerves, and blood vessels, the epidermis alone is the layer that constitutes the physical barrier towards ions, pathogens, and toxins within the environment (Rakers et al., 2010). Epidermal barrier function is provided by the epithelial cells (keratocytes), which are connected by a network of intercellular proteins known as tight junctions (TJs). TJs are composed of several transmembrane proteins, most notably claudin and occludin isoforms (Günzel and Fromm, 2012), which collectively regulate permeability and selectivity through the paracellular pathway (Chasiotis et al., 2012; Günzel and Yu, 2013).

The expression of claudin isoforms in fish skin has been shown to vary based on both body region (Gauberg et al., 2017) and environmental salinity (Bagherie-Lachidan et al., 2008; Bagherie-Lachidan et al., 2009). In relation to the latter, certain claudin-10 isoforms are thought to be associated with the formation of cation selective pores in seawater acclimated fish (Bui and Kelly, 2014; Marshall et al., 2018; Chen et al., 2021). In rainbow trout, the mRNA abundance of 10 different claudin isoforms was shown to vary in response to elevated cortisol (Gauberg et al., 2017), which is an important osmoregulatory hormone (Redding et al., 1984). This divergent expression of claudin isoforms likely contributes to differences in the permeability and selectivity of the paracellular pathway in fish skin. Goblet cell density and epidermal thickness have also been shown to vary in rainbow trout skin based following cortisol exposure (Gauberg et al., 2017), providing further evidence that the permeability and selectivity of the skin barrier may be physiologically regulated and may vary depending on body site.

The majority of knowledge related to ion transport and barrier function in fish skin stems from *in vitro* studies of the operculum, cleithrum or jaw skin - collectively referred to as cephalic skin (McCormick, 1994; Marshall and Bellamy, 2010; Glover et al., 2013). In teleost fish, mitochondria rich cells (MRCs) make up a significant proportion of both gill and cephalic skin epithelia (Foskett and Scheffey, 1982). Given the structural similarities between the two epithelia, the cephalic skin is suggested to be functionally similar to the gill, in which barrier and active transport functions have been extensively studied (McCormick, 1994; Glover et al., 2013). As such, it is unlikely that insights gained from studies of the cephalic skin are representative of trunk skin. Historically, the trunk skin has been cited as an impermeable barrier, incapable of electrogenic transport (Fromm, 1968). However, there is now growing evidence to suggest the trunk skin of teleost fish is capable of active ion transport. For example, the sodium-hydrogen antiporter (NHE), vacuolar type ATPase (V-ATPase), and Rhesus (Rh) proteins have all been identified in the trunk skin of rainbow trout (Zimmer et al., 2014). However, it has yet to be determined if these transporters contribute significantly to overall ion homeostasis (Wright and Wood, 2009).

The aim of this study was to assess and characterise the functional organization of the trunk skin barrier of rainbow trout and elucidate potential active transport mechanisms. To achieve this, two separate experiments were carried out. In the first experiment, barrier function and active transport were assessed in fully-intact skin (dermis, epidermis, and scales present) and in superficially wounded skin (scales removed). This allowed for the assessment of the contribution of each of these skin components to barrier function and active transport. Sampling at different time points following wounding also allowed barrier function and active transport to assessed at different points throughout the wound-healing process. In the second experiment, the contribution of the different skin layers to barrier function and active transport was further assessed in fully intact skin (dermis, epidermis, and scales present) and wounded skin (only dermis present). This experiment also measured regional differences in skin function, as well as the response of the skin to environmental variation in the form of salinity change. Finally, the second experiment assessed the effect of pharmacological inhibition on potential sites of active transport in the skin.

## Methods

### Experimental Fish and Holding Conditions

Rainbow trout (150–200 g) were obtained from Vänneån fish farm and maintained at the Department of Biological and Environmental Sciences, University of Gothenburg. Fish were maintained in 1 m^3^ concrete tanks supplied with freshwater (10°C) from a recirculating aquaculture system (RAS). For experiment two, a subset of fish was acclimated to SW by exchanging the inlet water from FW to 10°C artificial SW from another RAS so that full strength SW (35 ppt) was reached after approximately 24 h. This subset was maintained in SW for at least 2 weeks prior to commencing the experiment. All fish were maintained on a 12:12 day/night cycle and were fed commercial pellets (Biomar). All experimental procedures were approved by the Swedish Board of Agriculture (ethical permit number: 5.8.18-15096/2018). There were no mortalities reported during the experiment.

### Ussing Chamber Methodology

In both experiments, skin barrier function and active transport were assessed using the Ussing chamber technique (UCC-401; UCC-Laboratories Ltd.) described by Sundell et al. (2003), and using modifications described by Sundell and Sundh (2012). In brief, this method involves the measurement of several electrophysiological parameters from an isolated epithelium, which is simultaneously bathed by two independent solutions (one environmental/apical and one internal/basal). In both experiments, the skin was mounted in the chambers with the epidermis exposed to the environmental/apical chamber half, and the dermis exposed to the internal/basal chamber half. A gas mixture consisting of 99.7% air and 0.3% CO2 provided circulation, oxygenation and pH regulation to both chamber halves. This ensured viability of the excised skin for the duration of the experiment. The temperature of the chambers was kept at 10°C using a water-cooled mantle.

Alternating adaptive DC voltages (U) were applied to the mounted skin every 5 min using platinum electrodes. The applied voltages generated corresponding currents (I) varying between −30 and 30 μA, which prevented electrical charging of the epithelia. The U/I pairs assessed were fitted to a straight line using the least-square method. The slope of the line represented the transepithelial electrical resistance (TER). The short circuit current (SCC) was calculated where U = 0. A pair of KCl electrodes in a 3 M KCl solution continuously measured the transepithelial potential difference (TEP) across the epithelium *via* a series of agar bridges. The TER mainly reflects the paracellular shunt resistance created across the tight junction proteins between the epithelial cells, while the TEP reflects the net ion distribution across the epithelium as a result of the paracellular and transcellular transfer of ions. Finally, SCC is a measure of the net active ion transport across the epithelium.

### Experiment 1 – Skin Functions in Different Skin Tissue Layers and Healing of a Superficial Wound

FW acclimated rainbow trout (*N* = 31) were anaesthetised with FW buffered tricaine methanesulfonate (MS-222; 100 mg/L). The skin was superficially wounded by descaling an area (∼1.5 cm^2^) just below the dorsal fin using a scalpel. Fish were then allowed to recover in the concrete tanks before the skin was sampled. To sample the skin, the fish were euthanised in buffered tricaine methanesulfonate (MS-222; 250 mg/L), after which the gill arch was severed. Skin was sampled at the following time points: right after the wound (0 h), 3, 24, and 48 h post wounding. A dorsal-ventral incision was made posterior to the wound site. From here, two longitudinal incisions were made (one above the lateral line and one at the uppermost dorsal region), which extended towards the head. This resulted in a single strip of dorsal flank skin, roughly 3 cm wide, including the wounded area. This strip of skin was carefully separated from the underlying muscle by lifting and peeling with a scalpel. A section of intact skin was then sampled anterior to the wounded area. The excised skin was mounted in the Ussing chambers, with the epidermis facing the environmental side. Four millilitres of chilled Ringer’s solution (mmol L^−1^: NaCl 140, KCl 2.5, CaCl_2_ 1.5, MgSO_4_*7H_2_O 0.8, NaHCO_3_ 15, KH_2_PO_4_ 1, HEPES 5, D-Glucose 10, L-Glutamine 20) was added to both the apical and basal chamber halves, making the preparation isosmotic i.e. there was no concentration gradient to facilitate passive ion movement. A gas mixture consisting of 99.7% air and 0.3% CO_2_ provided circulation, oxygenation and pH regulation to ensure viability of the excised skin for the duration of the experiment. The temperature of the chambers was kept at 10°C using a water-cooled mantle. The skin was acclimated for 60 min.

After the acclimation period, Ringer’s solution containing ^14^C-mannitol (0.04 MBq ml^−1^) was added to the apical chamber half, while the basal side was also replenished with fresh Ringer’s solution. ^14^C-mannitol was used to assess epithelial permeability to small, uncharged molecules. After 20 min, 50 μl of solution was taken from both the apical and basal chamber halves. Additional 50 μl samples were taken from the basal side after 25, 30, 60, 80, 85 and 90 min. The samples were placed in scintillation vials filled with 4.5 ml UltimaGold (PerkinElmer, MA, United States). Radioactivity was determined in a beta counter (Wallac 1409 DSA Liquid Scintillation Counter; PerkinElmer, MA, United States). The apparent permeability was calculated as:

$$P_{app}=dQ÷dt\times 1\left(A\times Co\right)$$

Where dQ ÷ dt is the rate of radioactivity of the measured basal side (mol/s), A is the area of the Ussing chamber aperture, and Co (mol/ml) is the concentration of ^14^C-mannitol measured from the apical side. TER was also assessed for the duration of experiment 1, to determine the permeability of the skin to ion movement.

### Experiment 2

#### Effect of Body Region and Epidermal Loss on Skin Function

**FIGURE 1:**
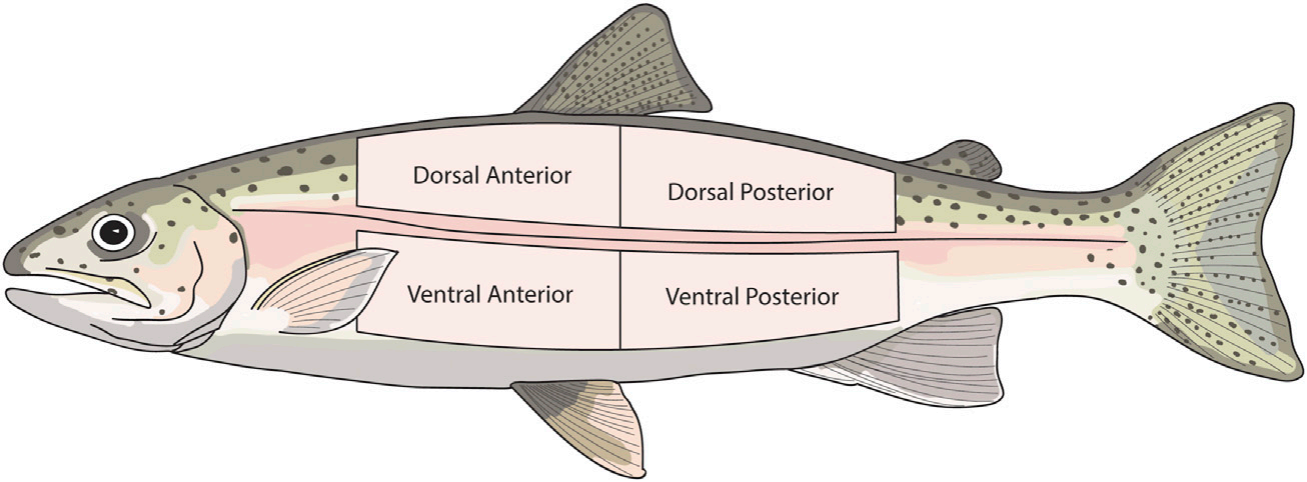
Diagram showing the skin regions that were sampled in experiment 2. The same skin regions were sampled from both the right and left side of each fish.

In experiment 2, the skin was sampled in the same manner as described in Section 2.3. However, to assess the effect of body region on skin function, skin was sampled from eight different locations on the body of each fish i.e. two dorsal and two ventral sections (posterior and anterior) on both the right and left side of each fish. To assess the contribution of the dermis to barrier function and active transport, one dorsal section and one ventral section from both the left and right side of each fish were experimentally damaged. This was achieved by scraping away both the epidermis and scales, leaving only the dermis. The damaged sections were alternated (anterior or posterior) between each fish. This resulted in four intact skin sections, and four damaged skin sections for each individual. The sampled regions are shown in Figure 1.

In total, 36 individuals were analysed, which comprised 288 skin sections. Once all of the skin sections were removed and prepared, they were mounted in their respective Ussing chambers as described in Section 2.3, with the difference that ^14^C-Mannitol was not used in experiment 2. FW Ringer’s solution (described in Section 2.3) or SW Ringer’s solution (mmol L^−1^: NaCl 150, KCl 2.5, CaCl_2_ 2.5, MgCl_2_*6H_2_O 1, NaHCO_3_ 7, NaH_2_PO4*2H_2_O 0.7, HEPES 5, D-Glucose 10, L-Glutamine 20) was added to each chamber half depending on acclimation salinity. The skin was then acclimated under isosmotic conditions for 60 min.

#### Effect of Salinity on Skin Function

To determine potential long-term and short-term responses to environmental salinity, four different salinity exposures were conducted. To explore barrier function and active transport in long-term acclimated fish, skin sampled from FW acclimated fish was exposed to FW, while skin from SW acclimated fish was exposed to SW. To explore the effect of acute salinity change on barrier function and active transport, skin from FW acclimated fish was exposed to SW, while skin from SW acclimated fish was exposed to FW. Each salinity exposure took place on the apical/epidermis side. To achieve this, the apical Ringer’s solution was replaced with 4 ml of either FW or SW after the 60 min acclimation period, while Ringer’s solution remained present in the basal chamber half. The basal Ringer’s solution was refreshed 110 min after commencing the experiment. In the first two series (FW-FW and SW-SW), 12 rainbow trout were analysed. Six individuals were analysed in each of the other series (FW-SW and SW-FW).

#### Effect of Pharmacological Inhibition on Skin Function

To characterise potential active transport, N-ethylmaleimide (NEM) was used to test for the presence of V-ATPase. This inhibitor was used as V-ATPase has previously been reported in rainbow trout skin (Zimmer et al., 2014). A stock solution of NEM (100 mM) was prepared using 70% ethanol as vehicle. Forty microliters of this stock solution was added to either the apical or basal chamber half, yielding a 1 mM concentration in the chambers. The chamber half that did not receive the inhibitor was supplied with 40 μl of vehicle only as the control. NEM was added 150 min after commencing the experiment. Once NEM was added, the experiment continued until the electrical parameters reached an approximate asymptote, hereafter referred to as steady state. When steady state was reached, the experiment was terminated.

### Immunohistochemistry

Immunohistochemistry was used to test for the presence of V-ATPase and NKA in the skin. Skin sections were excised from the same regions as experiment 2. The sections were fixed in 4% paraformaldehyde (PFA; 24 h), followed by decalcification in EDTA (0.5M; pH 8; 24 h). The samples were embedded in paraffin and sectioned (5 µm) using a rotary microtome (Shandon Finesse 325, Thermo Fisher Scientific, United States). The sections were attached to aminopropyltriethoxysilane (APES; Sigma A3648) coated slides. The slides were dewaxed using Histoclear and rehydrated *via* a graded ethanol series (100–70%) to distilled water, followed by tris-buffered saline and Tween 20 (TBST; 0.05M, pH 7.6, 2 × 5 min baths). The sections were bathed in a blocking buffer (5% normal goat serum, 5% normal donkey serum, 3% BSA in TBST) at room temperature for 60 min, followed by incubation in a humidity chamber overnight at 4°C with the primary antibody. The V-ATPase primary antibody (GenScript Cat. No. A00938) was diluted 1:1600 in blocking buffer, while the NKA (monoclonal mouse-anti NKA; α5) primary antibody was diluted 1:800. The slides were rinsed in TBST and incubated at room temperature with the secondary antibody. The V-ATPase secondary antibody (donkey anti-rabbit, Jackson ImmunoResearch) was diluted 1:200 in 0.05M TBST, while the NKA antibody (donkey anti-mouse, Jackson ImmunoResearch) was diluted 1:1000 in 0.05 M TBST. The slides were incubated with avidin-biotin-complex substrate (Vectastain ABC kit) for 30 min. Finally, the slides were stained with Vector NovaRed for 8–10 min, after which they were dehydrated *via* a graded ethanol series and mounted in Pertex mounting medium (Histolab Products AB, Sweden). The α5 antibody were developed by D. Fambourgh (John Hopkins University, Baltimore, MD) and was obtained from the Developmental Studies Hybridoma Bank developed under the auspices of the National Institute of Child Health and Human Development (NICHD; www.nichd.nih.gov) and maintained by the University of Iowa, Department of Biology, Iowa City, IA.

### Statistical Analysis

#### Experiment 1

Two-way ANOVAs were carried out to test the effect of superficial wounding on both TER and ^14^C-mannitol P_app_ (Log-transformed). For these tests, the main effects of wounding (intact/control and wounded) and time were tested. A possible significant interaction between the two was also tested. Tukey’s *post hoc* tests were carried out to assess which groups differed significantly.

#### Experiment 2

Welch tests were used to test for the effects of body region (dorsal-ventral, anterior-posterior, and right-left side) on TER and TEP under isosmotic conditions. Two-Way ANOVA was used to test for the effect of acclimation salinity and epidermal removal on TER (Log-transformed) and TEP under isosmotic conditions. To test for the effects of salinity, acclimation salinity and treatment salinity were combined into a new variable, hereafter referred to as salinity. This variable had four levels and is represented as “acclimation salinity-treatment salinity” (FW-FW, SW-SW, FW-SW, SW-FW). Welch’s ANOVA was used to test for the effect of salinity on TER, TEP, and SCC using the final measurement prior to pharmacological inhibition. *Post-hoc* comparisons were carried out for the ANOVA using Games-Howell tests. Welch tests were also used to test for significant differences in TER, TEP, and SCC for each salinity in response to NEM exposure. Here, the final steady state measurements were used. To visualise how TER, TEP and SCC changed over time, time series were plotted for random samples to show the acute effects of salinity alteration and NEM addition. All analyses were carried out in SPSS version 27 and Graphpad Prism version 9.

## Results

### Experiment 1 - Skin Functions in Different Skin Tissue Layers and Healing of a Superficial Wound

**FIGURE 2:**
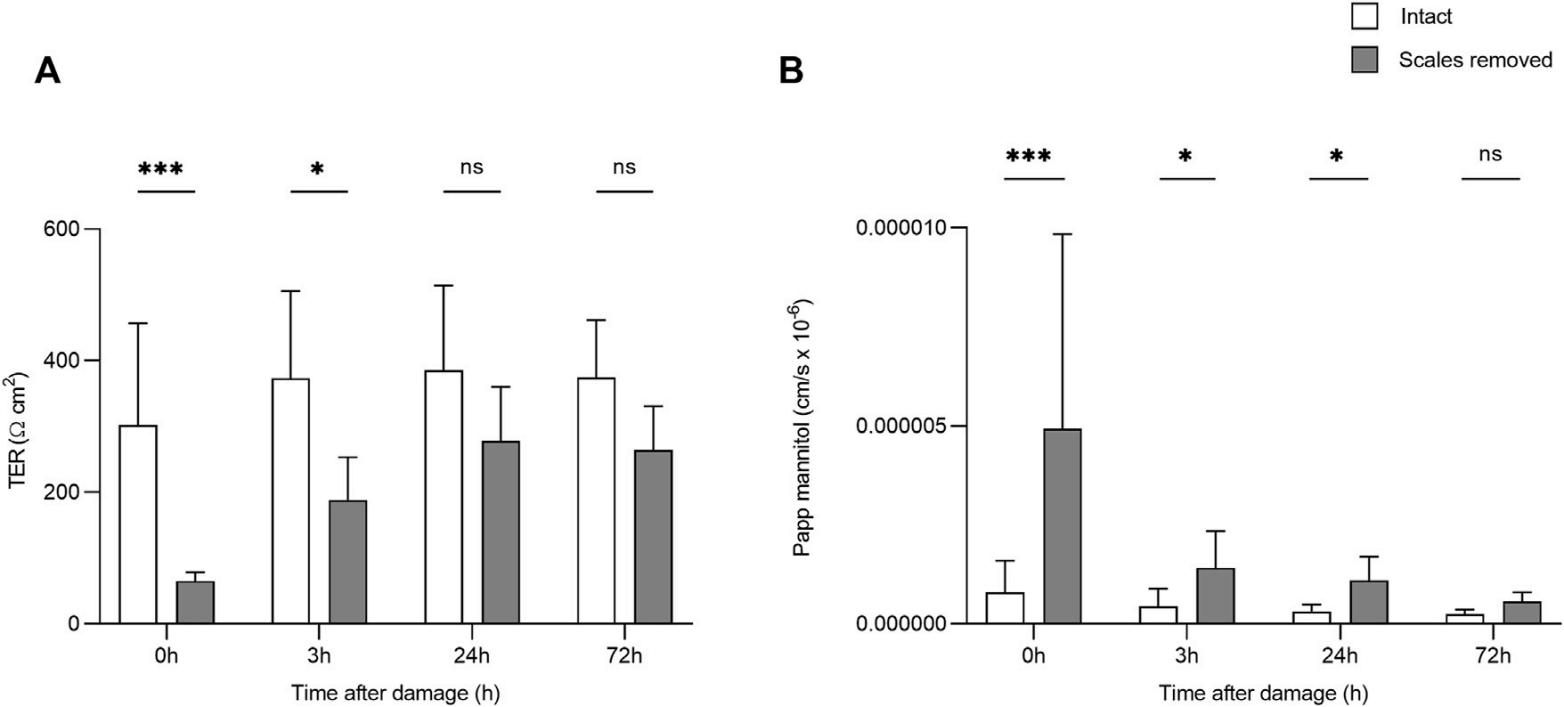
Bar chart showing **(A)** transepithelial resistance (TER) and **(B)** ^14^C-mannitol P_app_ (Log-transformed) in intact skin, and skin without scales but with intact dermis. Higher TER values indicate greater barrier function/lower transepithelial permeability, while higher ^14^C-mannitol P_app_ values indicate greater epithelial permeability. Bars represent mean and whiskers indicate standard deviation. Significant differences based on *post-hoc* Tukey tests are denoted with asterisks (***p < 0.001; *p < 0.05; ns, no significant difference).

After the creation of a superficial wound, TER varied significantly over time (F_3,50_ = 4.34, *p* = 0.009, η^2^ = 0.206) and based on damage (F_1,50_ = 38.10, *p* = <0.0001, η^2^ = 0.432; Figure 2). After 24 h, there was no significant difference in TER between the damaged and intact skin. ^14^C-mannitol P_app_ also varied significantly over time in the damaged skin (F_3,27_ = 11.076; *p* = <0.0001; η^2^ = 0.552), with ^14^C-mannitol P_app_ being significantly higher in the 0 h group compared to the 3, 24, and 72 h groups. Immediately after wounding (0 h), ^14^C-mannitol P_app_ was 83.6% higher in the damaged skin than in the intact skin. This had decreased to 68.7 and 71.7% after 3 and 24 h respectively. After 72 h, the difference had decreased to 56.6%, at which point the damaged and intact skin did not differ significantly. This reduction in permeability indicated the gradual formation of a neo-epidermis i.e. a newly formed epithelium. Significant differences in TER and ^14^C-mannitol P_app_ are shown in Figure 2.

### Experiment 2

#### Contribution of the Dermis to Skin Function and Variation Based on Body Region

**FIGURE 3:**
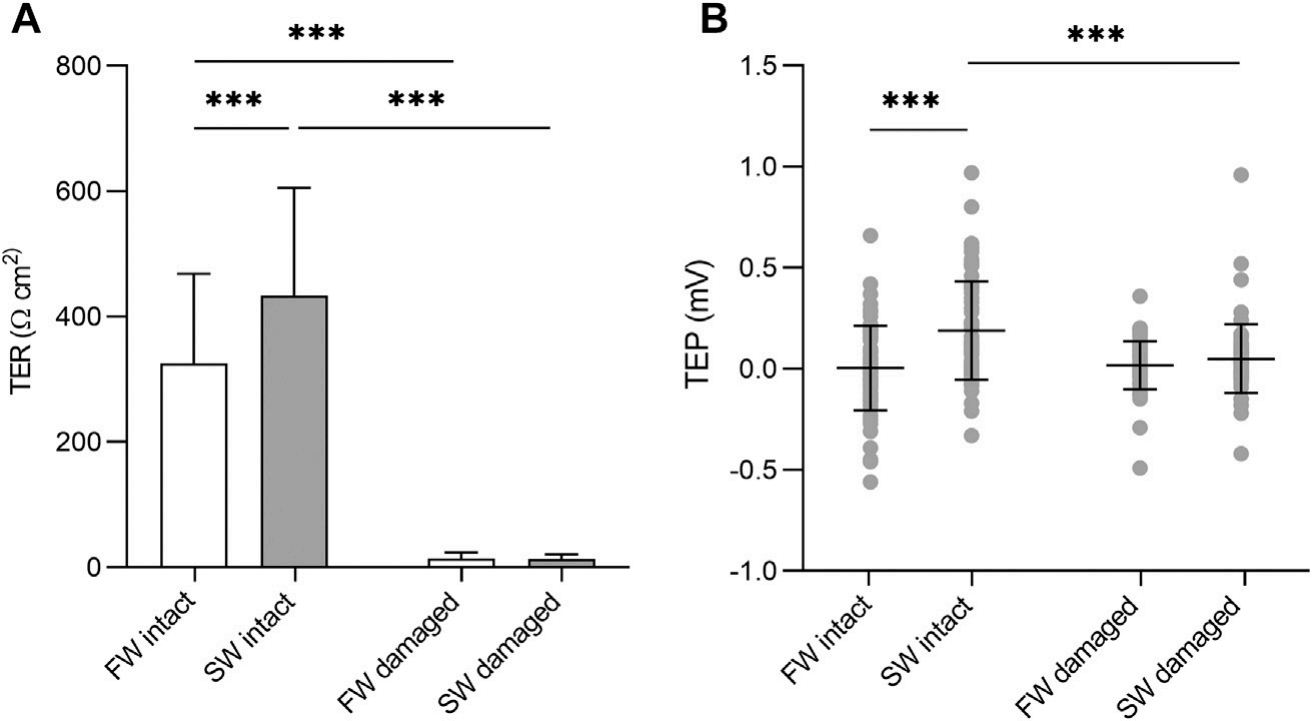
**(A)** Barchart of transepithelial resistance (TER) and **(B)** dotplot of transepithelial potential (TEP) in intact skin and skin without the epidermis. Bars represent mean and whiskers represent SD. Significant differences based on simple effects analysis using one –way ANOVA are denoted with asterisks (***p < 0.001).

There were no significant differences in TER or TEP based on body region (not shown). Under isosmotic conditions, complete epidermis/scale loss resulted in a 96 and 97% decrease in TER in the SW and FW acclimated fish respectively. The intact SW acclimated skin had significantly higher TER than the intact FW acclimated skin (F_1, 143_ = 23.87, *p* = <0.001) The TEP remained close to zero at isosmotic conditions, regardless of epidermal removal/acclimation salinity. However, there was a significant interaction between acclimation salinity and damage (F_1,284_ = 11.53, *p* = 0.039, η^2^ = 0.039). Subsequent analysis of simple effects showed that intact SW acclimated skin had a small but significantly higher TEP than intact FW acclimated skin (F_1,143_ = 23.87, *p* = <0.001) and damaged SW skin (F_1,143_ = 15.86, *p* = <0.001). Significant differences in TER and TEP are shown in Figure 3.

The ion content in the water directly affects the conductivity and hetero-osmotic solutions in each half chamber, resulting in passive ion potentials that affect the TEP. Based on the negligible changes in TER in the damaged skin (dermis only) following either FW or SW addition (Supplementary Figures S1, S2 respectively), the changes observed over time in the intact skin were clearly a physiological response of the epidermis. As the dermis did not appear to be functional in relation to either active transport or barrier function, only intact skin sections i.e. those not experimentally damaged were used for subsequent analyses.

#### The Effect of Salinity on Barrier Function and Active Transport

**FIGURE 4:**
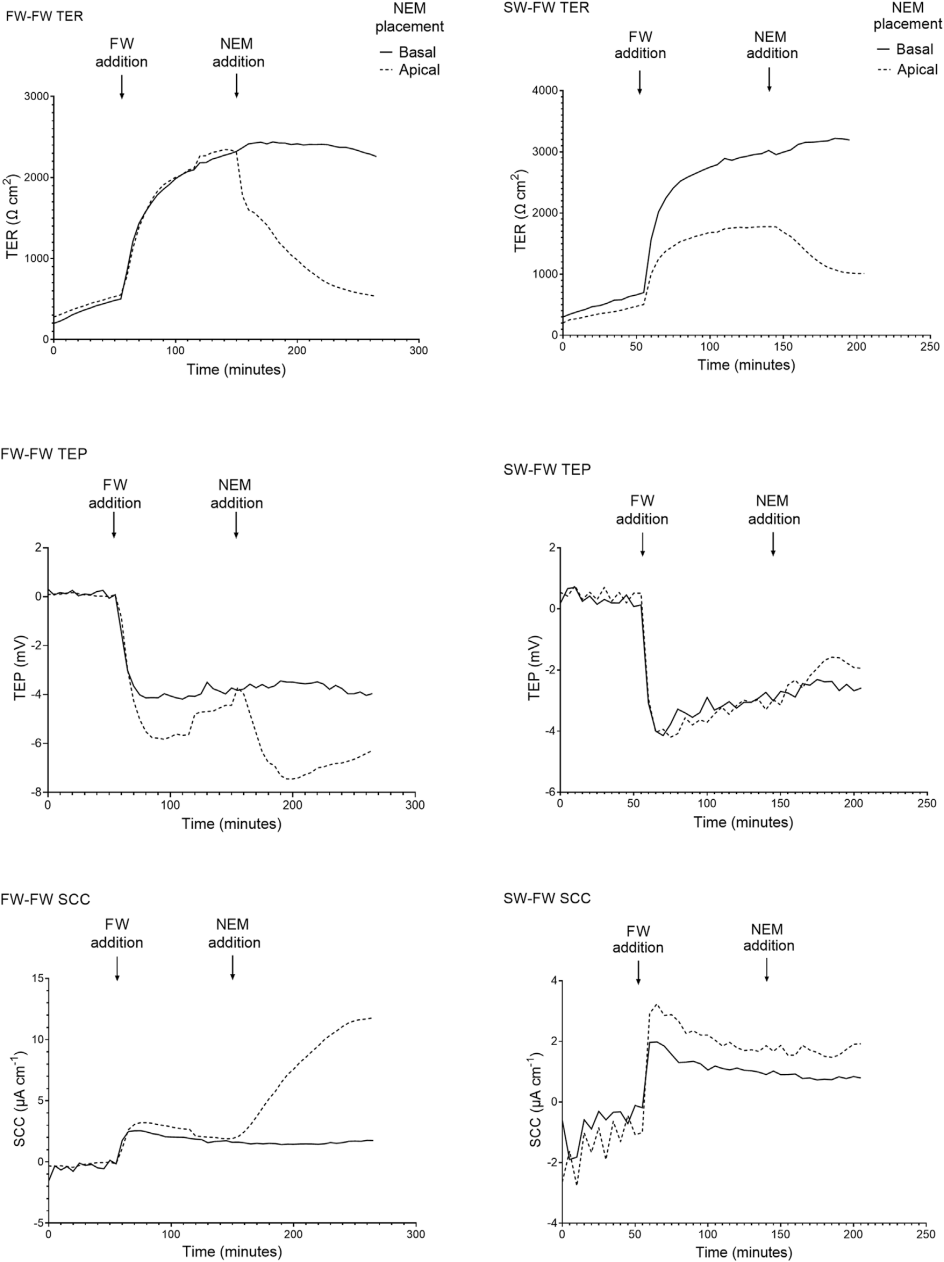
Representative traces of the electrical parameters transepithelial resistance (TER), transepithelial potential (TEP), and short circuit current (SCC) time series of fish exposed to FW (FW-FW and SW-FW) in the apical chamber half. A drastic increase in TER can be seen in the FW acclimated fish, accompanied by a reduction in TEP. The response to NEM addition is also shown, with a large reduction in TER and a reduction in TEP and SCC (most evident in the FW acclimated skin).

**FIGURE 5:**
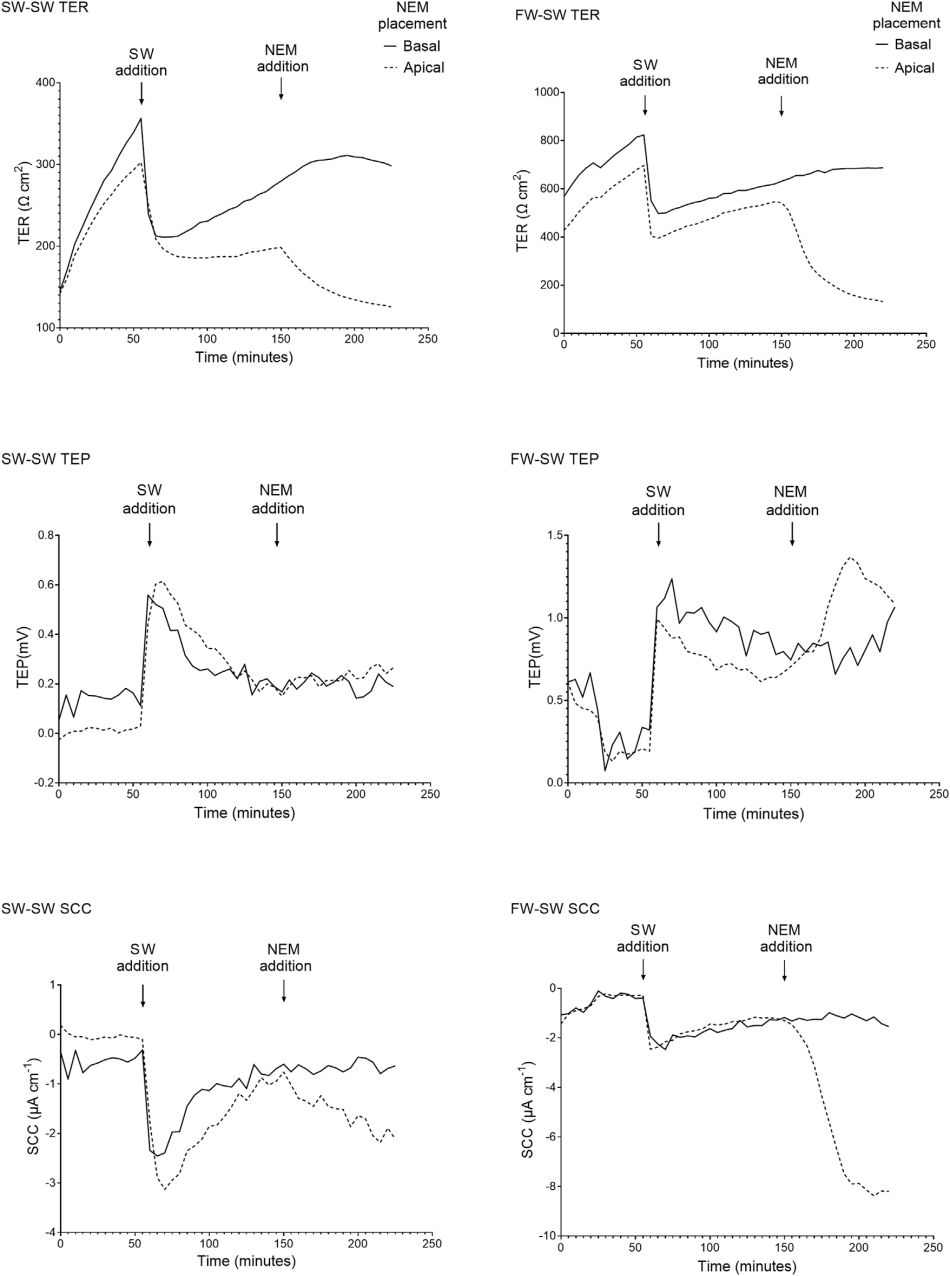
Representative traces of the electrical parameters transepithelial resistance (TER), transepithelial potential (TEP), and short circuit current (SCC) time series of fish exposed to SW (SW-SW and FW-SW) in the apical chamber half. A large and acute reduction in TER is shown following SW addition, accompanied by an acute change in TEP (basal-positive). The addition of NEM is also shown, with a reduction in TER and a change in TEP (basal-positive). Again, the changes in TEP and SCC following NEM addition are far more pronounced in the FW acclimated group.

When the apical Ringer’s solution was replaced with FW, the electrical parameters changed rapidly within minutes (Figure 4). This was accompanied by a large and rapid change in TEP (−4.2 ± 1.3 mV and −2.9 ± 1.4 mV respectively; basal-side negative) and SCC (3.6 ± 0.7 μA cm^−1^ and 1.9 ± 0.4 μA cm^−1^ respectively). Conversely, when SW was added to the apical chamber the TER instantly decreased, with the TER of the SW-SW and FW-SW groups averaging 301 ± 127 Ω*cm^2^ and 233 ± 140 Ω*cm^2^ respectively. This was accompanied by a slight increase in TEP (0.1 ± 0.3 mV and 0.02 ± 0.3 mV respectively; basal-side positive) and SCC (−0.3 ± 0.8 μA cm^−1^ and -0.5 ± 1.1 μA cm^−1^ respectively; Figure 5).

**FIGURE 6:**
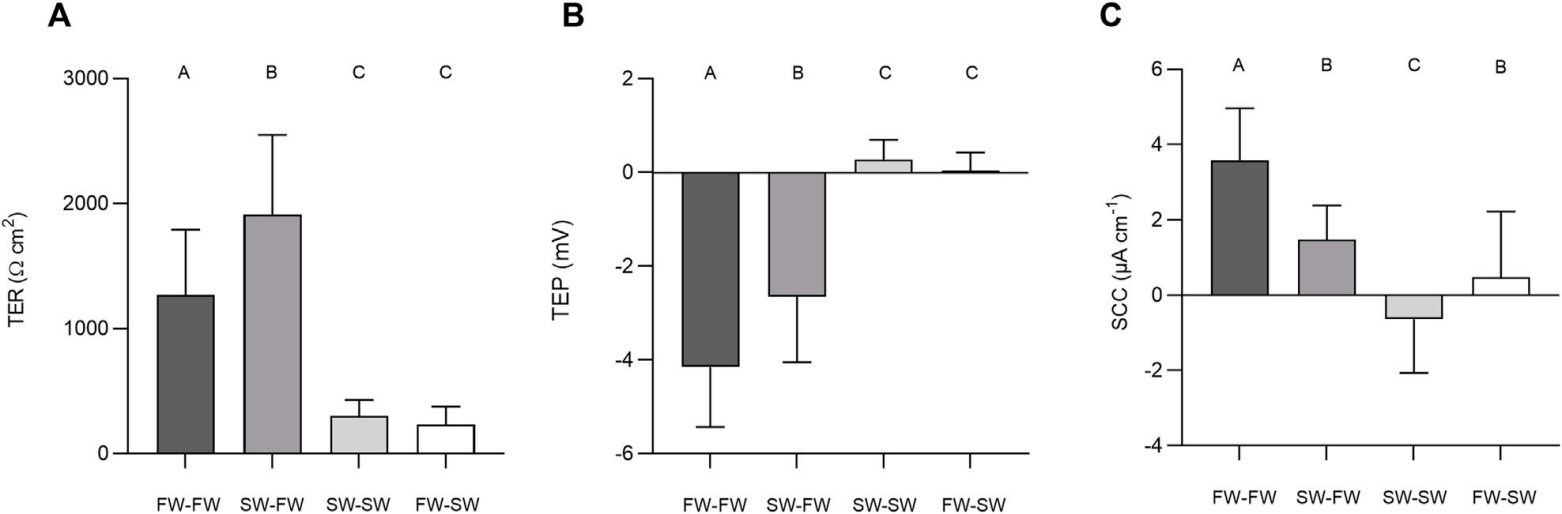
Barcharts showing the effect of salinity on **(A)** transepithelial resistance (TER), **(B)** transepithelial potential (TEP), and **(C)** short-circuit current (SCC). Bars represent means and whiskers represent SD. Groups not sharing a letter differ significantly based on the results of Welch’s ANOVAs and *post-hoc* Games-Howell tests.

The final TER, TEP, and SCC measurements before the addition of NEM were used to carry out separate Welch’s ANOVAs to assess the effect of salinity. Altering salinity in the apical chamber was found to significantly effect TER (F_3, 140_ = 118.40, *p* = < 0.0001, η^2^ = 0.717), TEP (F_3, 140_ = 199.47, *p* = < 0.0001, η^2^ = 0.810) and SCC (F_3, 140_ = 77.806, *p* = < 0.0001, η^2^ = 0.625; Figure 6). *Post-hoc* Games-Howell tests showed that each level within the factor “salinity” differed significantly from the other for TER and TEP, with the exception of SW-SW and FW-SW. For SCC, each level within the factor “salinity” differed significantly from the other, with the exception of SW-FW and FW-SW.

#### Pharmacological Inhibition of Primary Active Transport

**FIGURE 7:**
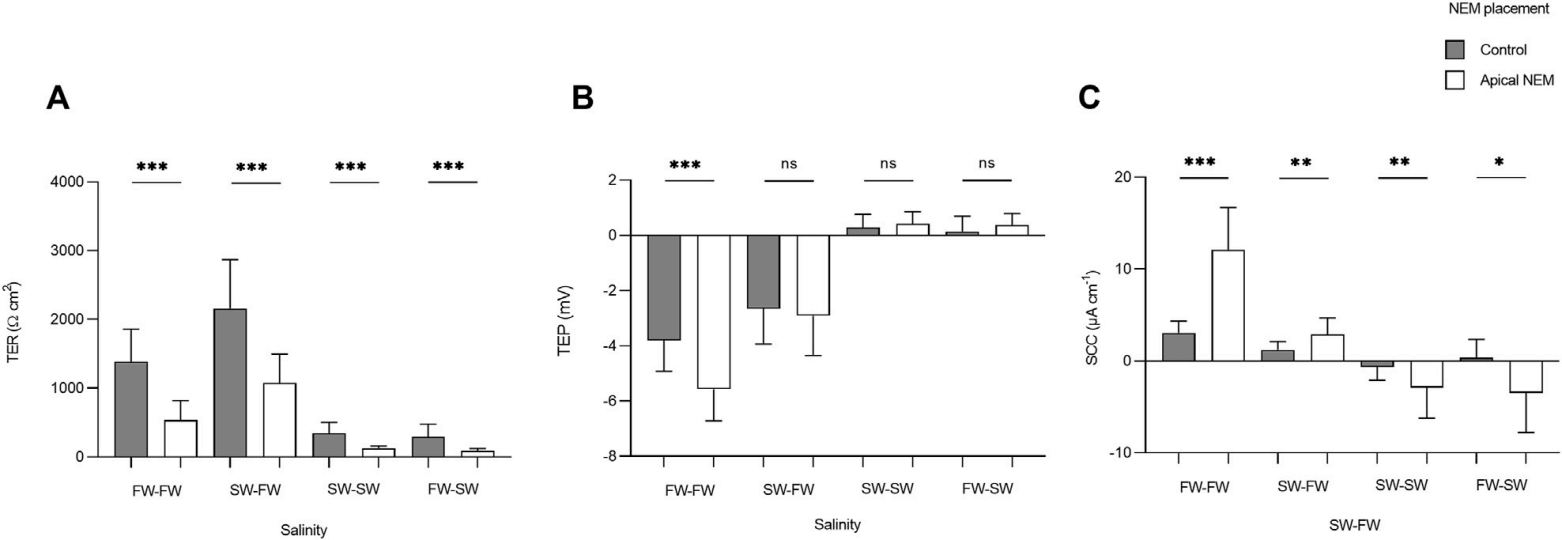
Barcharts showing the effect of basal (control) and apical N-ethylmaleimide (NEM) addition on **(A)** transepithelial resistance (TER), **(B)** transepithelial potential (TEP), and **(C)** short-circuit current (SCC) for each salinity. Bars represent means and whiskers represent SD. Significant differences based on Welch tests are denoted with asterisks (***p < 0.001; **p < 0.01; *p < 0.05, ns, no significant difference).

The addition of NEM induced a rapid reduction in TER, which was most evident in the FW acclimated fish. In the FW-FW group, this reduction in TER was accompanied by a similarly rapid and negative change in TEP. The effect of NEM addition on TER, TEP, and SCC was statistically tested for each level within the factor salinity (Figure 7). The addition of NEM to the apical chamber half significantly reduced TER in the FW-FW (F_1,46_ = 56.514; *p* = <0.001, η^2^ = 0.551), SW-FW (F_1,22_ = 20.734; *p* = <0.001, η2 = 0.485), SW-SW (F_1,46_ = 43.556; *p* = <0.001, η^2^ = 0.486) and FW-SW (F_1,22_ = 13.828; *p* = <0.001, η^2^ = 0.386) groups. Eta-squared values showed that the addition of NEM had the greatest effect on the FW-FW group, corresponding to a 58% reduction in TER compared to the control. The addition of NEM to the apical chamber half did not affect the TEP of any group, with the exception of FW-FW (F_1,46_ = 28.744; *p* = <0.001, η^2^ = 0.385). The addition of NEM also significantly affected SCC for each group: FW-FW (F_1,46_ = 89.658; *p* = <0.001, η^2^ = 0.661), SW-FW (F_1,22_ = 9.742; *p* = 0.005, η^2^ = 0.307), SW-SW (F_1,46_ = 9.770; *p* = 0.003, η^2^ = 0.175), and FW-SW (F_1,22_ = 7.628; *p* = 0.011, η^2^ = 0.257).

### Immunohistochemistry

**FIGURE 8:**
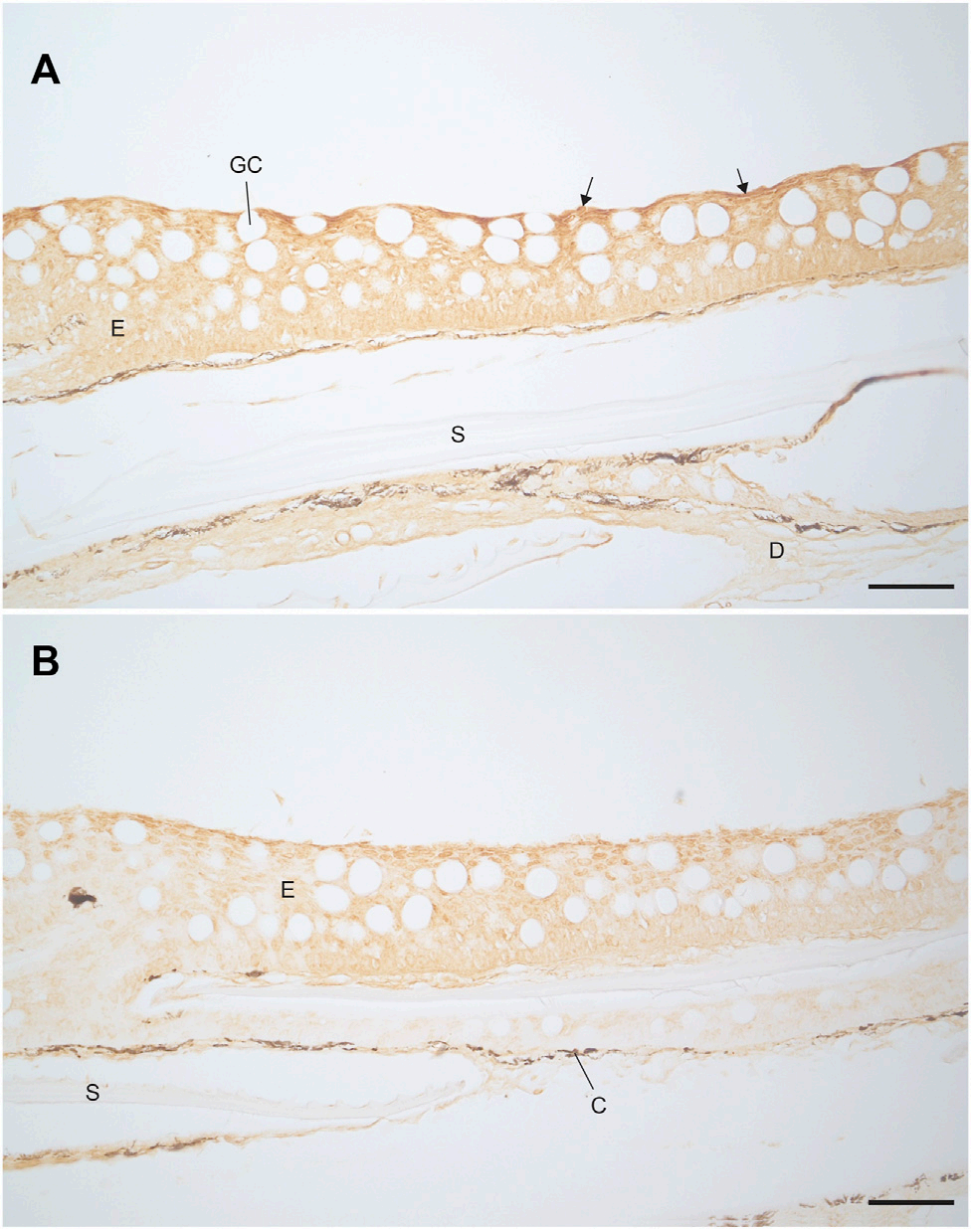
Localisation of V-ATPase in the trunk skin of **(A)** FW and **(B)** SW acclimated rainbow trout. Darker staining is visible along the apical epidermal cells of the FW fish (highlighted with arrows) compared to the SW fish. Scale bar = 50 µm. E, epidermis; D, dermis; GC, goblet cell; S, scale pocket; C, chromatophore.

Immunostaining with the V-ATPase antibody showed staining through the epidermis of both the FW and SW acclimated fish (Figure 8). The staining was stronger at the apical membrane for both FW and SW acclimated fish. This indicates the presence of V-ATPase in rainbow trout, regardless of acclimation salinity. However, the FW acclimated fish appeared to have stronger staining compared to the SW fish. Immunostaining with the NKA antibody showed no indication of NKA in either FW or SW acclimated fish (not shown), which indicates that MRCs were not present in the trunk skin.

## Discussion

The aim of this study was to assess and characterise the functional organization of the skin barrier of rainbow trout and to elucidate potential active transport mechanisms. To date, most studies into the electrophysiological properties of fish skin have focused on cephalic skin as a proxy for the gill (Glover et al., 2013), while the trunk skin has generally been overlooked as a site of both active transport and passive diffusion. In this study, we provide evidence to support the idea that rainbow trout skin is a dynamic tissue that quickly responds to salinity perturbations and is an active site of ion transport, functionally different from the branchial tissue and cephalic skin. We also confirm that the trunk skin creates a functional neo-epidermis within hours after wounding. In addition, we provide evidence to show that the epidermis is the main barrier forming component of the skin, while the dermis and scales play a negligible role in this regard.

### Skin Functions in Different Skin Tissue Layers and Healing of a Superficial Wound

The >95% loss of barrier function towards ions (TER) after removal of the epidermis and scales clearly showed that the dermis is not the rate limiting diffusion barrier of the skin. This was further demonstrated by comparing TER in the damaged and intact skin following acute salinity change. After altering salinity in the apical chamber half, the TER of the damaged skin changed only slightly, while the TER of the intact skin changed drastically. Further, the findings that the scales do not contribute significantly to barrier function, indicated as no significant difference between skin with scales and descaled skin with neo-epidermis, shows that the epidermis is the functional diffusion barrier in the skin. The dermis, in contrast to the epidermis, is composed mostly of fibrous connective tissue and is richly vascularised, which would structurally indicate a more leaky tissue layer (Rakers et al., 2010; Elliott, 2011). Thus, the epidermis is the main barrier between the environment and the underlying circulation. As such, any epidermal damage may lead to increased disease susceptibility and osmoregulatory problems that must be counteracted by the fish through energy demanding mechanisms (Sveen et al., 2020), which represents a threat to the health and welfare of the fish. In addition, damage to the epidermis may hinder the production of mucus, which is the first line of defence against adverse environmental conditions and pathogens (Shepherd, 1994; Benhamed et al., 2014; Kulczykowska, 2019). These results highlight the importance of minimizing skin damage in husbandry conditions.

The wound-healing experiment showed that scale/partial epidermal loss results in an almost complete loss of barrier function. However, significant healing or re-epithelialization appears to occur within 3 h. These results are consistent with histological studies of wound healing in *Salmo salar* (Roubal and Bullock, 1988; Sveen et al., 2019), *Bagarius bagarius* (Mittal and Munshi, 1974) *Cyprinus carpio* (Iger and Abraham, 1990), and *Danio rerio* (Richardson et al., 2013). In wounds where scales and epidermis are lost but the dermis remains, such as those used in the current study, the migration of epithelial cells occurs from the intact epidermis surrounding the wound and stops when the two fronts meet (Quilhac and Sire, 1999). The migration of epithelial cells has been shown to occur rapidly in cichlids and zebrafish, with a rate of 500 μm/h reported for both species (Quilhac and Sire, 1999; Richardson et al., 2016). However, the rate of re-epithelialization in rainbow trout is likely to be slower, given the much lower ambient temperature. This migration of epithelial cells and sealing of the epidermal layer provides a functional barrier while the scales regrow (Richardson et al., 2016; Sveen et al., 2020). In salmonids, scale remodelling begins 14 days post-wound and continues for around 36 days, with the rate of scale regrowth depending on several factors including diet, temperature, and stocking density (Jensen et al., 2015; Sveen et al., 2018). Although re-epithelialization is a fast process, scale loss as a result of husbandry practices will result in acute barrier failure in the short term. For this reason, scale loss should be considered an indication of severely impaired welfare.

Interestingly, the scales themselves do not appear to provide a significant diffusion barrier for ions or molecules. The plausible explanation for this is that the scales are embedded and surrounded by the extracellular matrix of the dermis, which in itself is leaky and clearly does not provide an adequate diffusion barrier to either ions or small inert molecules such as mannitol. In addition, the scale are separated from each other and hence do not constitute a continuous layer of tissue. However, as scales are composed of both a mineralised layer and an overlapping fibrous, mineralized collagen layer, they serve as a protective, armour-like barrier against physical trauma (Elliott, 2011). Thus, even though descaled skin is quickly covered by a neo-epidermis, the wounded area is still very sensitive to physical trauma.

### Regional Differences in Skin Functions and Effect of Salinity

There was no difference in electrical parameters of the trunk skin based on body regions investigated. This is consistent with Zimmer et al. (2014), who found no differences in body region in rainbow trout trunk skin regarding ammonia transport. The lack of regional variation in any electrical parameter is interesting as Gauberg et al. (2017) previously showed differences in claudin expression along the dorsoventral axis of rainbow trout trunk skin. However, claudins are not only present in the TJs, but are also important in normal cell-cell interactions outside of the TJs. In the mammalian skin, claudins are expressed throughout the stratified epithelium of the epidermis, while functional TJs including claudins, occludins and ZO-1 are mainly formed in the cell layer just underneath the keratinized dead layer (Brandner, 2009). Thus, variations in claudin expression may also reflect general cell-cell contact in the stratified epithelium of fish skin.

Acute exposure to FW or SW, rather than long-term salinity acclimation, showed that trunk skin has the ability to respond to salinity changes, with increased TER being observed following FW exposure. This is consistent with responses observed in cephalic skin (Marshall et al., 1992), and cultured rainbow trout gill epithelia (Wood and Pärt, 1998; Wood et al., 2002). While the physiological mechanism behind the acute change in skin permeability in response to salinity is unknown, there are several possibilities. First, the selectivity and permeability of the paracellular pathway in epithelia is determined by the characteristics of the tight junctions (Mills et al., 1977; Bäsler et al., 2016), and in particular by the claudin protein family (Kolosov et al., 2013).

In fish skin, claudin expression has been shown to vary in response to environmental salinity, with the expression of certain claudin-10 isoforms increasing following SW acclimation in several species (Bui and Kelly, 2014). This is consistent with studies of the gill of several teleost species, where SW transfer also increases the expression of claudin-10 isoforms (Tipsmark et al., 2008a, Tipsmark et al., 2008b, Tipsmark et al., 2016; Bossus et al., 2015) and with the opercular skin of *Fundulus heteroclitus* (Marshall et al., 2018; Chen et al., 2021). Certain claudin-10 isoforms are thought to provide the paracellular pathway with cation selective pores, which facilitate ion efflux in SW (Marshall et al., 2018). However, Kolosov et al. (2020) recently demonstrated that claudin-10 is downregulated following SW acclimation in the lamprey, indicating that the contribution of claudins to epithelial barrier function may be species dependent.

Conversely, the decrease in epithelial permeability observed in the current study following exposure to FW may be related to the incorporation of barrier forming claudins into the TJs. Studies into claudin expression in *tetraodon nigroviridis* skin (Bagherie-Lachidan et al., 2008; Bagherie-Lachidan et al., 2009) and intestine (Clelland et al., 2010) suggested that an increase in claudin-3 abundance was associated with reduced permeability, while an increase in claudin-27a abundance following SW acclimation was associated with reduced permeability. In FW *O. mykiss* skin, claudin-30 is the most abundantly expressed isoform, with claudins-28b and -31 also expressed abundantly (Gauberg et al., 2017). Claudin-30 has been functionally characterized in *Salmo salar* where it forms a barrier towards Na^+^ (Engelund et al., 2012). In addition, claudins-28b and -31 have been shown to increase in abundance following transfer from SW to FW in *Oreochromis mossambicus* (Tipsmark et al., 2016), suggesting a possible role in reducing epithelial permeability. This pattern is consistent with observations in cultured *O. mykiss* gill pavement cells, where claudin-28b abundance is drastically reduced following hyperosmotic stress (Sandbichler et al., 2011).

Another possible explanation to the differences in barrier function between FW and SW could lay in the morphological arrangement of the TJs. The length of TJs can be broadly categorised into shallow or deep, based on the relative depth of the protein strands comprising the TJs (Engelund et al., 2012). Shallow TJs have been reported in the gills of SW acclimated fish (Sardet et al., 1979; Karnaky, 1986). These shallow TJs are much leakier than the deep TJs found in FW acclimated fish (Sardet et al., 1979) and primary gill cell cultures exposed to FW, which also display deep TJs (Wood and Pärt, 1997). Thus, the acute changes in TER observed in the current study in response to salinity changes may be related to a rapid remodelling of the TJs, where the TJ complexes increase and decrease in depth following exposure to FW and SW respectively. Similar changes in TJ structure have been shown to occur rapidly in cultured branchial epithelia after alteration of salinity (Wood et al., 2002; Chasiotis et al., 2012). As such, it is possible that similar morphological changes occur in the skin TJs. The exact mechanisms of TJ remodelling in teleost fish are unclear. However, there is ample evidence from mammalian cells to suggest that the TJs are dynamic and capable of rapidly remodelling in response to changes in external stimuli through phosphorylation and/or endocytosis of TJ proteins (Van Itallie and Anderson, 2006; Shen et al., 2008; Stamatovic et al., 2017). Such responses to external stimuli have been shown to occur over minutes (Gonzalez et al., 2008).

Finally, it is possible that the mucus layer may have contributed to the observed differences in barrier function between FW and SW exposed skin. The mucus provides the first layer of protection to the fish and acts as a direct defence against environmental contaminants. However, there is also evidence to suggest that mucus composition is salinity dependent, with SW acclimated fish having more viscous mucus, which is related to differences in mucus hydration (Roberts and Powell, 2005). The salinity-dependent nature of mucus also appears to hold true for the skin, with Ordóñez-Grande et al. (2020) showing increased mucus exudation in hyperosmotic conditions. In addition, Ordóñez-Grande et al. (2021) suggested a possible role of skin mucus as a layer to trap ions in hyposmotic conditions while the facilitating water retention in hyperosmotic conditions, thus playing an important role in osmoregulation. Further studies are needed to elucidate the role of rainbow trout skin mucins in barrier function during salinity acclimation.

### Evidence for Active Transport in the Trunk Skin

A pronounced basal-negative TEP and corresponding change in SCC quickly developed after the exchanging the isosmotic Ringer’s solution with FW to the apical chamber of the FW acclimated group. The parameters stabilized after approximately 30 min at ∼ −4mV and ∼3.6 μA/cm^2^. The levels were stable for the remainder of the experiment, suggesting the involvement of at least one active transporting component. Similar responses in TEP have been observed in salmonid cephalic skin and gill primary cell cultures (Marshall et al., 1992; Wood and Pärt, 1997; Wood et al., 2002). A negative basal solution can be the result of a net movement of negative ions from the apical to the basal side, or a net movement of positive ions from the basal to apical side. The latter is supported by strong immunoreactivity of V-ATPase in the apical part of the epidermis of FW acclimated fish.

These findings are in agreement with the localization of a V-ATPase in the FW gill pavement cells of rainbow trout (PVCs; Sullivan et al., 1995). In the FW gill, apical H+ extrusion by V-ATPase drives the uptake of Na+ from the environment through a phenamil sensitive Na+ channel (ENaC; Reid et al., 2003). Interestingly, in SW acclimated trout gill, V-ATPase abundance and activity decreases 70–80% compared to FW (Hawkins et al., 2003) and H+ secretion is instead achieved by an apical NHE driven by the inwardly directed Na+ gradient (Evans et al., 2005).

An attempt to elucidate the role of the V-ATPase in trunk skin was made by inhibiting skin V-ATPase using NEM, a highly potent and specific inhibitor (Lin and Randall, 1995). Addition of NEM to the apical but not basal chamber half resulted in changes in TEP and SCC (basal-negative), indicating that V-ATPase may be one component of the active transport in rainbow trout trunk skin. However, the apical addition of NEM also resulted in reduced TER. This suggests that NEM has the capacity to increase paracellular permeability. The ability of NEM to increase TJ permeability has been demonstrated in toad bladder (Marples et al., 1994) and Madin-Darby canine kidney (MDCK) cells (Kanda et al., 2018). Thus, in addition to inhibiting the apical V-ATPase, NEM also increases ion permeability across the TJs, which may facilitate passive ion diffusion due to the difference in ion composition between the two chamber halves. As NEM contributes to changes in both active transport and passive diffusion across the skin, the observed changes in TEP and SCC following NEM addition cannot be assigned solely to inhibition of the V-ATPase, but is probably also a result of increased paracellular permeability due to disrupted tight junctions.

### CONCLUSIONS

In this study, we assessed the contribution of the different skin layers to barrier function, determined the effect of salinity, and tested for possible active transport mechanisms. The epidermis was found to be the diffusion barrier in the skin, with the scales and dermis playing a negligible role. Epidermal barrier function was mainly determined by environmental salinity, with FW exposure resulting in a strong reduction of epithelial permeability. While the rapid salinity-dependent changes in skin function are likely related to acute changes in TJ structure and function, the exact mechanisms behind the acute changes in epithelial permeability remain unclear. However, the salinity dependent differences in barrier function observed in the current study are consistent with previous studies that have showed varying expression of TJ proteins in the skin of fish. In relation to active transport, further research is required to determine if skin V-ATPase contributes significantly to overall ion homeostasis in rainbow trout, or whether it plays a more specialised role.

## Data Availability

The raw data supporting the conclusions of this article will be made available by the authors, without undue reservation.
