# Supplementary material for: Transport and Barrier Functions in Rainbow Trout Trunk Skin Are Regulated by Environmental Salinity
Source: Front Physiol. 2022 May 13;13:882973. doi: 10.3389/fphys.2022.882973 (PMC9136037; doi:10.3389/fphys.2022.882973)
Supplement: Supplementary file 1 [file Table1.DOCX]

Supplementary material


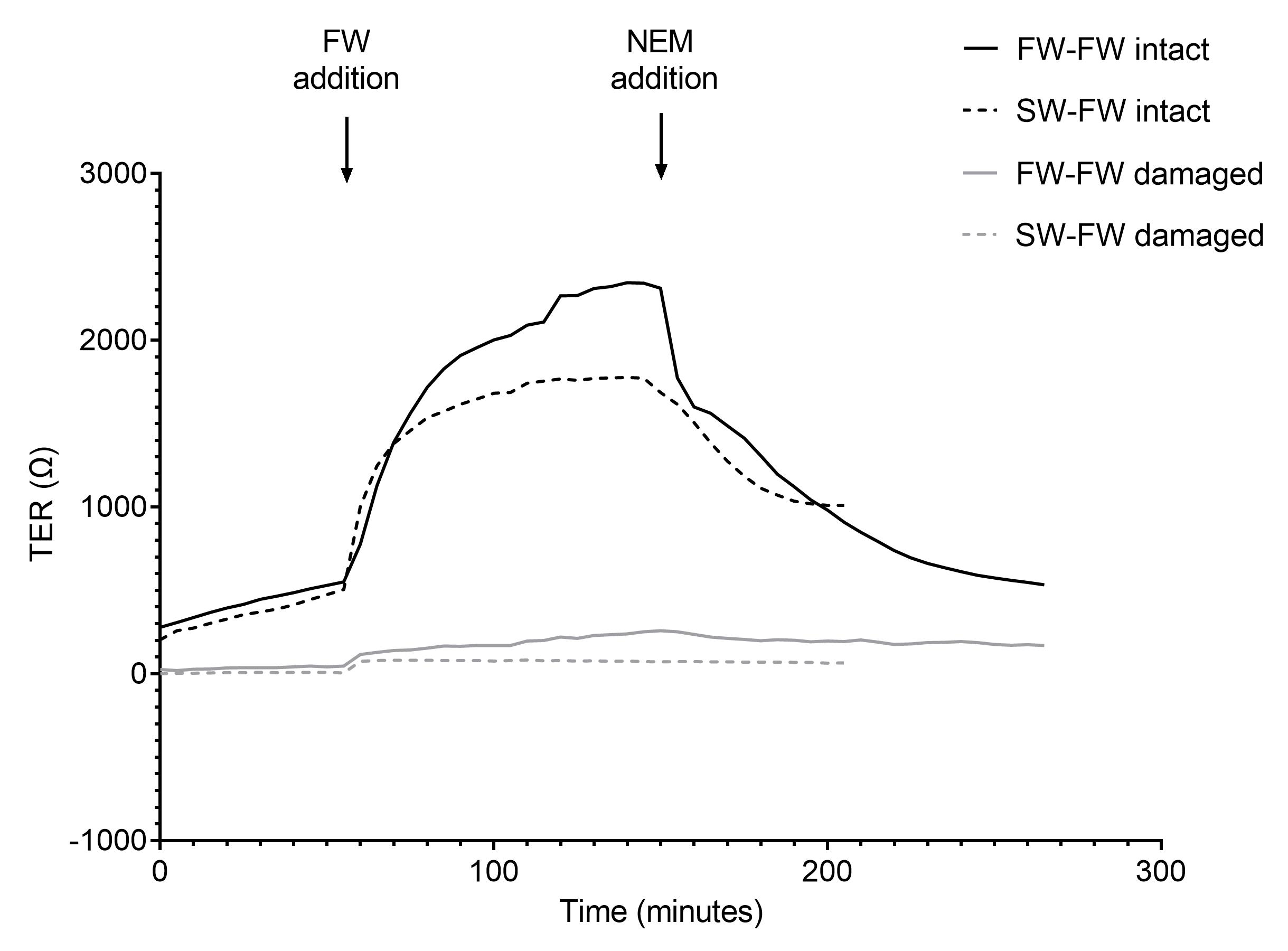


Figure S1. Representative trace of transepithelial resistance (TER) in intact and experimentally damaged skin. A drastic increase in TER can be seen following the addition of FW in the intact skin. Conversely, the damaged skin shows only a very small change in TER. Similarly, the damaged skin does not appear to have any response to the addition of NEM.


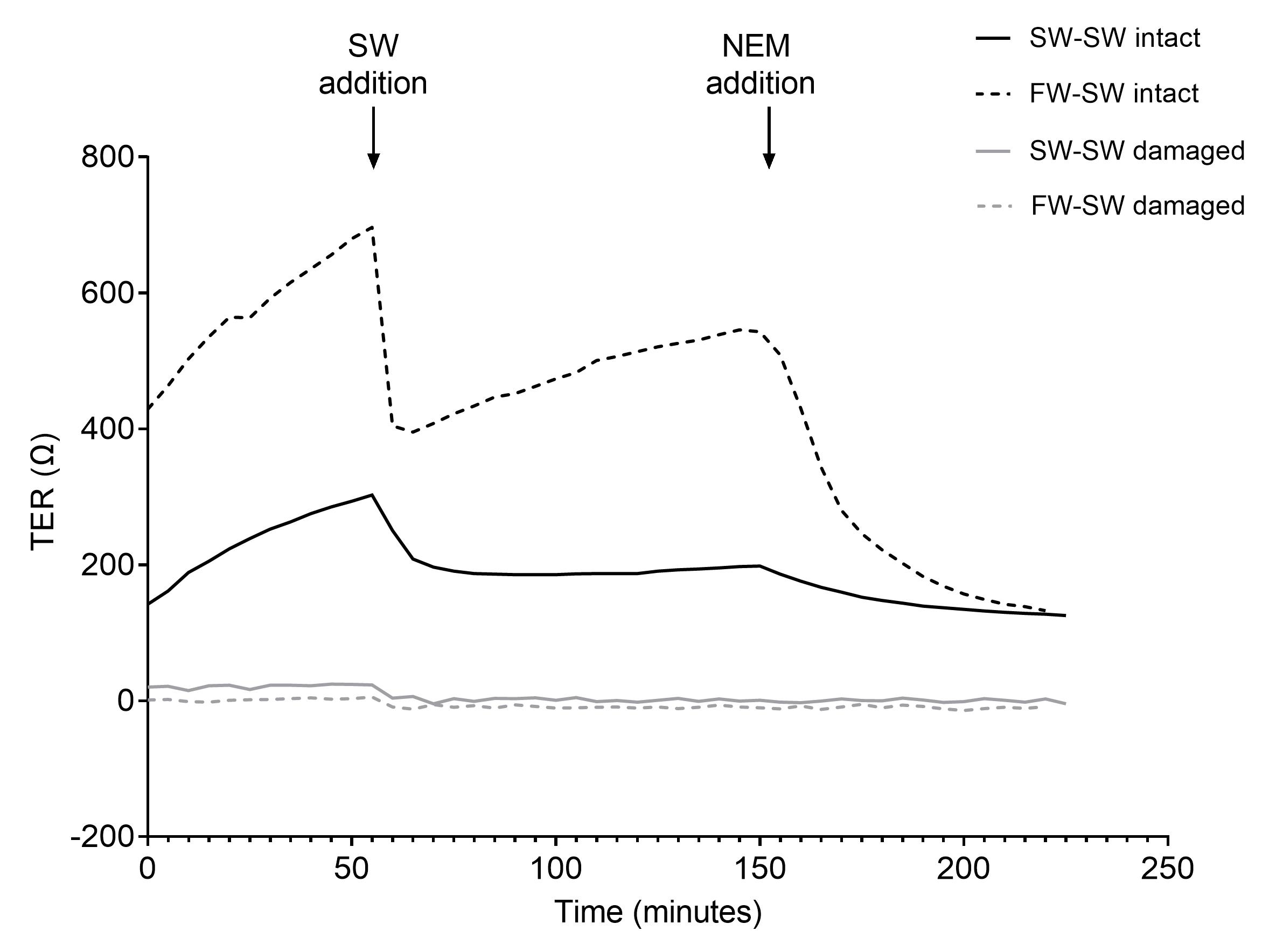


Figure S2. Representative trace of transepithelial resistance (TER) in intact and experimentally damaged skin. A drastic decrease in TER can be seen following the addition of SW in the intact skin. Conversely, the damaged skin shows only a very small change in TER. Similarly, the damaged skin does not appear to have any response to the addition of NEM.
